# Editorial: Memristor Computing for Neuromorphic Systems

**DOI:** 10.3389/fncom.2021.755405

**Published:** 2021-09-08

**Authors:** Kyeong-Sik Min, Fernando Corinto

**Affiliations:** ^1^School of Electrical Engineering, Kookmin University, Seoul, South Korea; ^2^Department of Electronics, Politecnico di Torino, Turin, Italy

**Keywords:** memristors, neuromorphic, in-memory computing, neural networks, beyond von Neumann architecture

Recently, traditional computing systems based on the von Neumann architecture are facing the very well-known problems of memory-access bottleneck and energy efficiency wall. The memory bottleneck is because of separation of computing and memory blocks in the computing architecture. The energy efficiency wall is caused from the end of device and voltage scaling. Moreover, amounts of data collected from Internet-of-Things (IoT) sensors are exploding day by day for threatening computing capability in terms of computing energy and performance. To address all these issues, new computing hardware needs to be developed, which can be based on extreme-parallel architecture, brain-inspired or brain-mimicking for realizing synaptic plasticity, capable of in-memory computing, suitable for analog arithmetic, multi-values logic, etc. Memristors have been studied very intensively because they are useful for realizing a new computing hardware satisfying the conditions mentioned above. Memristors are non-volatile memory devices. These devices are very energy-efficient and fast during write and read operations. Memristor crossbars are fabricated in a CMOS-compatible process and can compute analog arithmetic. These features make in–memory computing architecture promising for the realization of neuromorphic systems and the other brain-mimicking ones. In the future we can think that such systems could be very suitable for solving the problems of memory access bottleneck and energy efficiency wall mentioned above.

To reflect the research interests mentioned above, the following five papers were carefully reviewed and finally published in this Research Topic. The detailed contribution of each paper is explained one by one as below.

Taking inspiration from the previous scientific related developments (Lanza et al.), report, through the Flux-Charge Analysis Method (FCAM), that the state space in the (i–v)-domain can be decomposed in many invariant manifolds, where the system may exhibit different attractors. Therefore, this leads that bifurcations can be induced both by initial conditions on a fixed manifold (as in the classical theory of dynamical systems), or, most important for the purpose of this paper, by changing the invariant manifold. Finally, in the paper, it is analyzed how the creation of time-varying long-range connections and the recurrent switch of invariant manifold lead to an improvement of the network synchronization properties.

Kim et al. report a spiking-based simulator that works with unsupervised learning. Using this simulator, the authors perform the simulation of SNN behaviors with a hardware-based synaptic model having a non-linear weight update. In the simulation, unlike DNN, which achieves the highest performance when the weight update curve is linear, SNN is more influenced by the symmetry properties and balance of potentiation and depression weight update curve. This finding may be a useful indicator for implementing a hardware based SNN system by testing elements of various characteristics.

Ascoli et al. applies the techniques from Theory of Local Activity to the strongly-non-linear dynamics of a niobium dioxide (NbO) micro-scale threshold switch fabricated at the facilities of NaMLab gGmbH. The micro-scale device, falling into the class of first-order voltage-controlled generic volatile memristors, exhibits a Negative Differential Resistance (NDR) region on its DC current-voltage characteristic. Importantly, this device is able to amplify infinitesimal fluctuations in energy, which is a signature for its capability to enter the locally-active (LA) operating mode. Gaining a deep understanding of the conditions necessary for polarizing the NaMLab memristor in the LA domain can be of primary importance for the later adoption of the device in bio-inspired circuit design.

Recently, memristor circuits have been observed that they are able to generate coexistence of multiple complex dynamics, which can be activated by impulsive external inputs. Based on this observation (Innocenti et al.), investigate the basic mechanisms which can be exploited to make a very simple memristor circuit mimicking a spiking or bursting neuron. Possibly, this result can lead to design micro-circuits naturally acting as real neuronal nets.

Choi et al. report an RRAM-based hybrid synaptic circuit which is composed of a 'big' synapse and a “small” synapse. Moreover, they propose a related training method for the hybrid synaptic circuit. Unlike the previous programming methods, array-wise fully-parallel learning can be performed successfully with the proposed architecture having a simple array selection logic, as explained in the paper. To experimentally verify the operation of the proposed hybrid synapse, the authors test the proposed synaptic circuit with Mo/TiOx RRAM, which shows promising synaptic properties and areal dependency of conductance precision. Through neural network simulations, they verify that the proposed RRAM-based hybrid synapse circuit and the related learning method can achieve maximum accuracy as large as 97%, comparable to the software implementation which has the accuracy as large as 97.92% when each device has only 50 conductance states.

## Summary

This Research Topic has collected the results of research and developments on memristor-based neuromorphic and in-memory computing. More specifically, this special topic tries to deal with various techniques using algorithms, applications, systems, circuits, memristive devices, etc., for realizing neuromorphic and in-memory computing systems, in which CMOS and memristors can be fabricated together to process vast amounts of unstructured data from numerous Internet-of-Things sensors. The collection of papers in this topic can be helpful to advance memristor-computing-based neuromorphic techniques further, in order to predict future potential computing hardware which can be based on beyond-Von-Neumann computing with new emerging memories such as memristors.

## Author Contributions

Both authors have contributed to the submitted manuscript.

## Conflict of Interest

The authors declare that the research was conducted in the absence of any commercial or financial relationships that could be construed as a potential conflict of interest.

## Publisher's Note

All claims expressed in this article are solely those of the authors and do not necessarily represent those of their affiliated organizations, or those of the publisher, the editors and the reviewers. Any product that may be evaluated in this article, or claim that may be made by its manufacturer, is not guaranteed or endorsed by the publisher.

